# The accuracy of four formulas for LDL-C calculation at the fasting and postprandial states

**DOI:** 10.3389/fcvm.2022.944003

**Published:** 2022-08-18

**Authors:** Jin Xu, Xiao Du, Shilan Zhang, Qunyan Xiang, Liyuan Zhu, Ling Liu

**Affiliations:** ^1^Department of Cardiovascular Medicine, The Second Xiangya Hospital, Central South University, Changsha, China; ^2^Research Institute of Blood Lipid and Atherosclerosis, Central South University, Changsha, China; ^3^Modern Cardiovascular Disease Clinical Technology Research Center of Hunan Province, Changsha, China; ^4^Cardiovascular Disease Research Center of Hunan Province, Changsha, China; ^5^Department of Gastroenterology, The Second Xiangya Hospital, Central South University, Changsha, China

**Keywords:** LDL-C, postprandial, Friedewald formula, Vujovic formula, Martin–Hopkins formula, Sampson formula, vertical auto profile method

## Abstract

**Background:**

Elevated level of low-density lipoprotein cholesterol (LDL-C) is concerned as one of the main risk factors for cardiovascular disease, in both the fasting and postprandial states. This study aimed to compare the measured LDL-C with LDL-C calculated by the Friedewald, Martin–Hopkins, Vujovic, and Sampson formulas, and establish which formula could provide the most reliable LDL-C results for Chinese subjects, especially at the postprandial state.

**Methods:**

Twenty-six subjects were enrolled in this study. The blood samples were collected from all the subjects before and after taking a daily breakfast. The calculated LDL-C results were compared with LDL-C measured by the vertical auto profile method, at both the fasting and postprandial states. The percentage difference between calculated and measured LDL-C (total error) and the number of results exceeding the total error goal of 12% were established.

**Results:**

The calculated LDL-C_F_ levels showed no significant difference from LDL-C_VAP_ levels at the fasting state. The calculated LDL-C_S_ were significantly higher than LDL-C_VAP_ at the fasting state (*P* < 0.05), while the calculated LDL-C_s_ were very close to LDL-C_VAP_ levels after a daily meal. At the fasting state, the median total error of calculated LDL-C_F_ was 0 (quartile: −3.8 to 6.0), followed by LDL-C_S_, LDL-C_MH_, and LDL-C_V_. At the postprandial states, the median total errors of LDL-C_S_ were the smallest, 1.0 (−7.5, 8.5) and −0.3 (−10.1, 10.9) at 2 and 4 h, respectively. The calculated LDL-C_F_ levels showed the highest correlation to LDL-C_VAP_ and accuracy in evaluating fasting LDL-C levels, while the Sampson formula showed the highest accuracy at the postprandial state.

**Conclusion:**

The Friedewald formula was recommended to calculate fasting LDL-C, while the Sampson formula seemed to be a better choice to calculate postprandial LDL-C levels in Chinese subjects.

## Background

Cardiovascular disease (CVD) has been the leading cause of death worldwide (1). The elevated level of low-density lipoprotein cholesterol (LDL-C) is concerned as one of the main risk factors of CVD, especially for atherosclerotic CVD (2). In clinical practice, it is the main laboratory parameter used for cardiovascular risk assessment and the primary target for cholesterol control (3). Therefore, it is crucial to ensure reliable measurement of LDL-C levels.

There are several methods to measure LDL-C levels, including ultracentrifugation, formula methods, direct method, and nuclear MR (NMR) method (4). Among them, the ultracentrifugation method is recommended as the reference method for LDL-C measurement. Due to complex operations and expensive equipment, ultracentrifugation is difficult to be widely used in clinical practice. Vertical auto profile (VAP) methodology, one of the ultracentrifugation methods, was used to measure lipid profiles as a reference method, commonly at the fasting state (5). However, a study involving the measurement of lipid profiles by VAP at the postprandial or non-fasting state is very rare worldwide (6, 7), and there was no similar study in China.

In comparison, the formula method is simpler and cheaper. The Friedewald method developed in 1972 is the main mathematical formula for LDL-C calculation (8). It uses a fixed coefficient, 2.2 (for mmol/l), to describe the relationship between triglyceride (TG) and very-low-density lipoprotein cholesterol (VLDL-C) (8). When the TG level was above 4.5 mmol/l, the accuracy of this formula will decline. Thus, other formulas were proposed. In 2003, the Vujovic formula, which uses 3.0 (for mmol/l) as a ratio of TG to VLDL-C, was proposed for LDL-C calculation (9). Then, the Martin–Hopkins formula was developed with an adjustable ratio based on TG and non-high-density lipoprotein cholesterol (non-HDL-C) levels (10). In 2020, Sampson and colleagues (11) proposed a new formula, which was proved to be suitable for samples with TG levels up to 9.0 mmol/l. Those novel formulas were proved to be more accurate than the Friedewald formula (12).

Recently, a variety of expert recommendations have supported non-fasting lipid assessment (13–15), as elevated non-fasting TG and LDL-C levels had been regarded as independent risk factors of atherosclerotic CVD (16, 17). We once reported that the direct measured LDL-C levels were significantly higher than calculated LDL-C levels by the Friedewald formula at both the fasting and non-fasting states in Chinese subjects (18). Three novel formula methods and the VAP method were not involved in this study. Thus, this study aimed to establish which formula method could provide the most reliable LDL-C results when compared with the VAP method for Chinese subjects, especially in the postprandial state.

## Methods

### Study subjects

There were 26 subjects (in-patient) included in this study in the Department of Cardiovascular Medicine of the Second Xiangya Hospital, Central South University. All the subjects were invited to fill out a questionnaire on medical history and use of medication before participation. Subjects with fasting TG levels above 4.5 mmol/l were excluded. No subject had a history of thyroid diseases, liver and kidney diseases, autoimmune diseases, cancer, or other severe medical illnesses. The study was approved by the Ethics Committee of the Second Xiangya Hospital of Central South University and informed consent was gained from all the participants.

### Specimen collection

After at least 12 h of overnight fasting, venous blood samples were collected in all the subjects before (i.e., 0 h) and at 2 and 4 h after taking a daily breakfast according to their daily habits, such as steamed bread, rice porridge, or noodles (19). All the subjects were required to complete the meal in 15 min. All the blood samples were centrifuged at 4°C for 3,000 rpm for 15 min and stored at −80°C refrigerator until analysis.

### Laboratory assays

Blood lipids were detected in two ways. First, all the blood samples were measured in a medical laboratory in Second Xiangya Hospital by a laboratory technician who had no knowledge of this study as described before (20). Serum levels of total cholesterol (TC) and TG were measured by automated enzymatic assays, and the concentration of HDL-C was measured by a direct method, i.e., the selective protection method. LDL-C level was measured directly by the chemical masking (CM) method (i.e., LDL-C_CM_) regardless of TG level. Then, the VAP method was used to measure all the lipid profiles, including LDL-C (i.e., LDL-C_VAP_), as a reference method (5). In brief, it simultaneously measures cholesterol concentrations of all the five lipoprotein classes in <1 h. After centrifugation, the contents of the centrifuge tube (separated layers of lipoproteins) were analyzed for cholesterol using the continuous flow VAP analyzer (5).

### Low-density lipoprotein cholesterol calculation

For each sample, the LDL-C level was calculated using mathematical formulas with CM measured lipids as follows:

Friedewald (8): LDL-C_F_ = TC – HDL-C – TG/2.2 (mmol/l)

Vujovic (9): LDL-C_V_ = TC – HDL-C – TG/3 (mmol/l)

Martin–Hopkins (10): LDL-C_MH_ = TC – HDL-C – TG/adjustable factor (mg/dl)

Sampson (11): LDL-C_S_ = TC/0.948 – HDL-C/0.971 – (TG/8.56 + TG × non-HDL-C/2,140 – TG2/16,100) – 9.44 (mg/dl).

The factor of 0.026 was used to convert LDL-C from mg/dl into mmol/l, if necessary.

### Statistical analysis

All the continuous levels were expressed as median (interquartile range) and qualitative variables were expressed as numbers and percentages. The measured TC, HDL-C, LDL-C, and the calculated LDL-C were tested to be distributed normally, while the measured TG was proven to be non-normal distribution. The parametric and non-parametric statistical tests were used for corresponding data, respectively. Differences between different groups were analyzed by *one-way* ANOVA, while differences among different time points within the same group were analyzed by repeated measure *one-way* ANOVA analysis. Categorical variables were compared using the *chi-squared* statistic tests. The Bland–Altman difference plots were used to compare calculated LDL-C levels and the LDL-C_VAP_. Correlation between calculated LDL-C levels and the LDL-C_VAP_ was conducted with Pearson correlation analyses. For each sample, the total error (%) between the mathematically calculated LDL-C and LDL-C_VAP_ was estimated as follows: [(LDL-C_formula_ – LDL-C_VAP_)/LDL-C_VAP_] × 100%. The accuracy of estimation was defined as the total error ± 12% (21). All the statistical analyses were performed with SPSS version 25.0. All the *P*-levels were 2-tailed, and *P* < 0.05 was considered statistically significant. For differences between LDL-C_VAP_ and LDL-C_formula_, *P* < 0.01 was considered statistically significant, as we replaced *post-hoc* analysis in the repeated measure one-way ANOVA analyses using one-way ANOVA analyses.

## Results

### Population characteristics

There were 26 subjects who participated in this study, including 17 (65.4%) men and 9 (34.6%) women. Their ages ranged from 46 to 73 years, with a median age of 62.5 years. Five of them got a body mass index (BMI) of over 28 kg/m^2^ and the median BMI of all the subjects was 25.5 kg/m^2^. The patients with coronary heart disease, hypertension, and diabetes accounted for 69.2, 69.2, and 30.8%, respectively. There were 42.3% of smokers and 46.2% of subjects taking statins (Table 1).

**Table 1:** Study population characteristics.

**Parameters**	***N* = 26**
Male, *n* (%)	17 (65.4)
Age, y	62.5 (54.75, 66.5)
BMI, Kg/m^2^	25.51 (23.2, 27.0)
Current smoking, *n* (%)	11 (42.3)
CHD, *n* (%)	18 (69.2)
Hypertension, *n* (%)	18 (69.2)
Diabetes, *n* (%)	8 (30.8)
History of statins, *n* (%)	12 (46.2)

Values are represented as median (interquartile range) and n (%) as appropriate.

BMI, body mass index; CHD, coronary heart disease.

### Postprandial changes in serum levels of blood lipids measured by different methods

**Figure 1:**
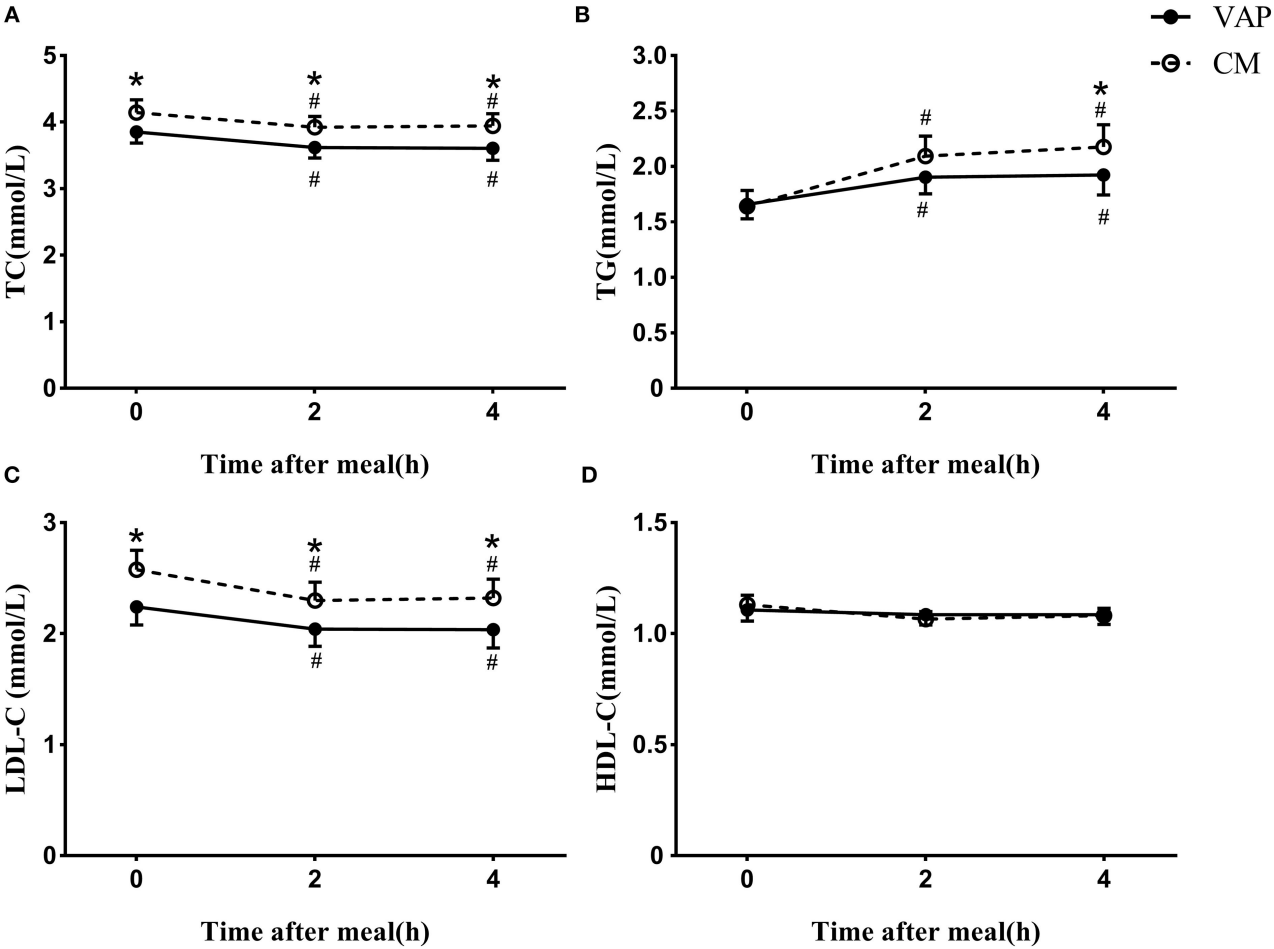
Changes in serum levels of blood lipids via VAP and chemical masking method after a daily meal. *P < 0.05 when compared with VAP measured values at the same time point. ^#^*P* < 0.05 when compared with fasting value using the same measure method.

It was obvious that the levels of TC, TG, and LDL-C measured by CM were significantly higher than those measured by VAP, while HDL-C levels measured by the two methods were similar at both the fasting and postprandial states (Figure 1). No matter which method was used, both the TC and LDL-C levels decreased significantly after a daily meal compared to the fasting state (*P* < 0.05, Figures 1A,C), while TG showed tremendously increase at the postprandial time points (*P* < 0.05, Figure 1B). The levels of HDL-C kept stable after a daily meal no matter which method was used (Figure 1D).

**Figure 2:**
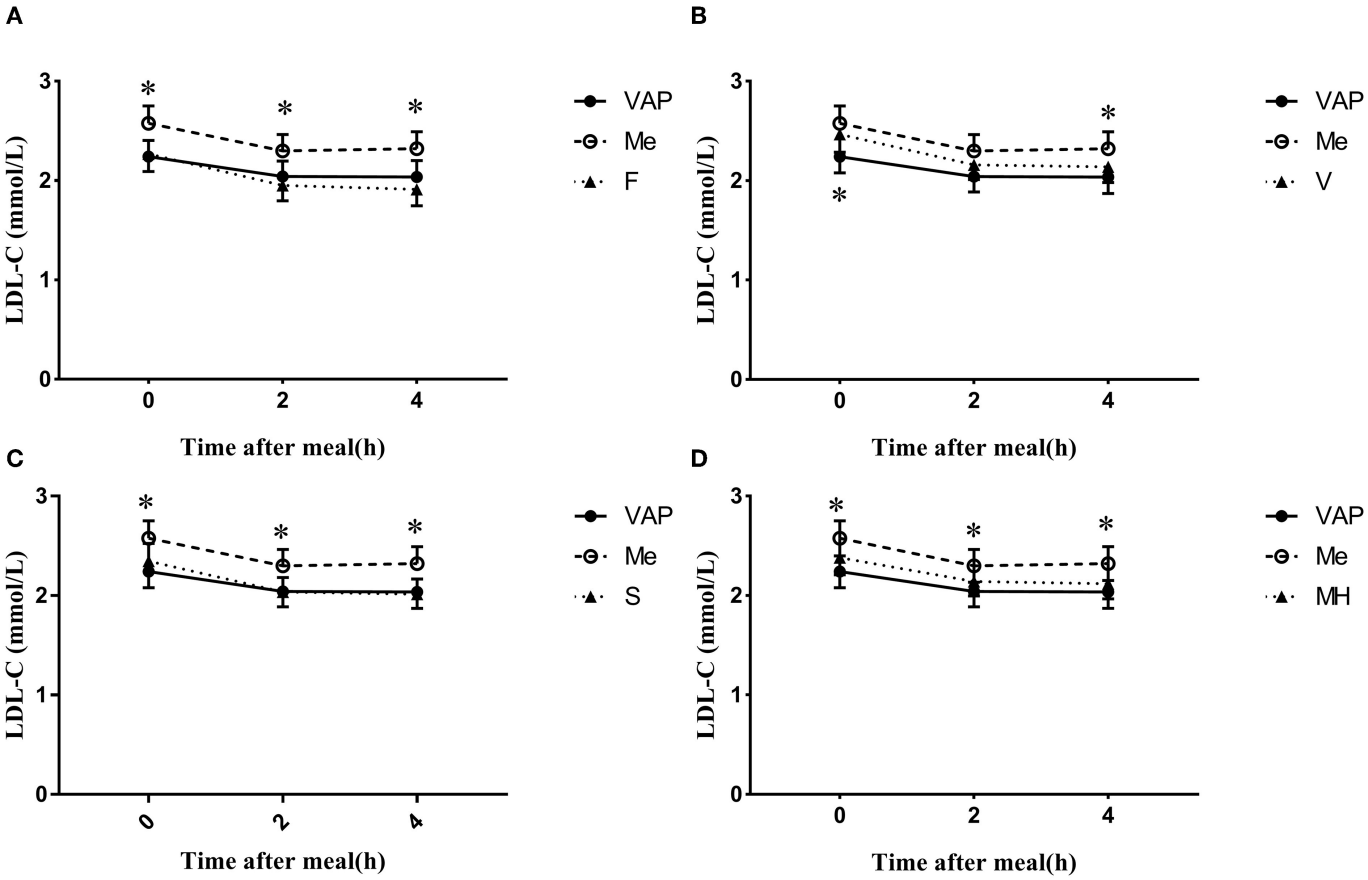
Changes in LDL-C levels via different calculated methods after a daily meal. *P < 0.05 when compared with calculated values at the same time point.

The calculated levels of LDL-C were acquired by four different formulas with blood lipids measured by CM, and they showed a similar decrease after a daily meal and the postprandial changes reached a statistic difference (*P* < 0.05, Figure 2). It is worth noting that there was no significant difference between calculated LDL-C levels at 2 and 4 h, no matter which formula was used.

The calculated LDL-C levels *via* Friedewald, Martin–Hopkins, and Sampson formulas showed no significant difference when compared to LDL-C_VAP_ levels at both the fasting and postprandial states (Figures 2A,C,D). However, the calculated LDL-C_V_ levels were significantly higher than LDL-C_VAP_ levels at the fasting state (*P* < 0.05 Figure 2B), while the calculated LDL-C_V_ levels were very close to LDL-C_VAP_ levels after a daily meal (Figure 2B). The calculated LDL-C_F_ levels seem to be lower than LDL-C_VAP_ levels at the postprandial states, while the difference did not reach statistical significance (*P* < 0.05, Figure 2A).

The calculated LDL-C_F_, LDL-C_S_, and LDL-C_MH_ levels were all significantly lower than LDL-C_CM_ levels at the fasting and postprandial states (*P* < 0.05, Figures 2A,C,D). The LDL-C_V_ levels were lower than LDL-C_CM_ levels; however, the difference reached statistical significance only at 4 h postprandially (Figure 2B).

### Consistency and correlation between estimated and measured low-density lipoprotein cholesterol

The Bland–Altman difference plots showed great consistency in estimated and measured LDL-C (Supplementary Figure 1). The measured LDL-C_CM_ also had a good consistency with LDL-C_VAP_ (Supplementary Figure 1). The Pearson correlation analyses showed a strong and positive correlation between LDL-C_VAP_ levels and the calculated LDL-C levels by four formulas, and the *r* levels ranged from 0.836 to 0.961 at the fasting and postprandial states (*P* < 0.05, Table 2). At the fasting state, the strongest correlation was found between LDL-C_VAP_ levels and LDL-C_F_ levels (*r* 0.870, *P* < 0.05). At the postprandial states, the strongest correlation was found between LDL-C_VAP_ levels and the LDL-C_V_ levels (2 h: *r* 0.961, 4 h: *r* 0.956, *P* < 0.05).

**Table 2:** Correlation in chemical masking method measured and formula estimated vs. VAP measured LDL-C.

	**LDL0**	**LDL2**	**LDL4**
Friedewald	0.870	0.920	0.913
Vujovic	0.85	0.961	0.956
Martin/Hopkins	0.837	0.927	0.902
Sampson	0.836	0.939	0.928
CM	0.780	0.883	0.859

A positive correlation was also found between LDL-C_VAP_ and LDL-C_CM_ at the fasting and postprandial states (0 h: *r* 0.780, 2 h: *r* 0.883, 4 h: r 0.859, *P* < 0.05, Table 2). However, the correlation between LDL-C_VAP_ and LDL-C_CM_ was weaker than the correlation between LDL-C_VAP_ and the calculated LDL-C levels by four formulas at both the fasting and postprandial states (Table 2).

### Distribution of the total error at the fasting and postprandial states

**Figure 3:**
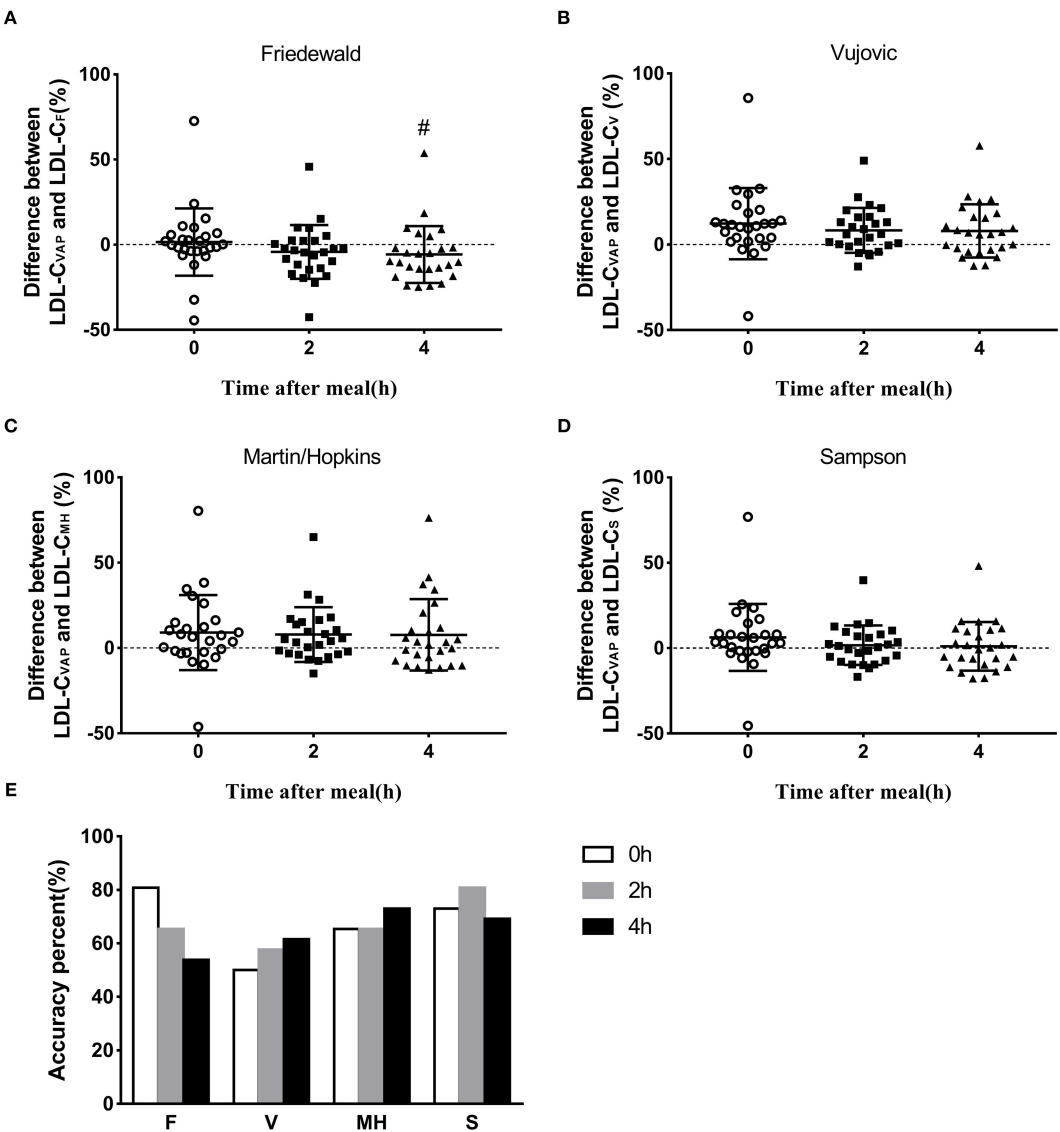
Accuracy of estimated LDL-C via different formulas. **(A–D)** Difference between LDL-C_VAP_ and LDL-C_formula_ at fasting and postprandial states. **(E)** Accuracy percent (total error ±12%) of calculated value via four formulas at fasting and postprandial states. ^#^*P* < 0.05 when compared with fasting state.

To determine the reliability of calculated LDL-C levels by different formulas, we calculated the total errors between calculated LDL-C and LDL-C_VAP_ levels. At the fasting state, the median total error of calculated LDL-C_F_ was 0 (quartile: −3.8 to 6.0; Figure 3A; Supplementary Table 1). The median total errors of calculated fasting LDL-C_V_, LDL-C_MH_, and LDL-C_S_ were 11.2 (3.2, 18.9), 7.0 (−2.5, 15.3), and 3.4 (−1.7, 10.0), respectively (Figures 3B–D; Supplementary Table 1).

The median total errors of LDL-C_F_ were −3.9 (−14.1, 2.4) and −9.9 (−15.3, 0) at 2 and 4 h, respectively (Figure 3A; Supplementary Table 1), which suggested that the Friedewald formula could underestimate LDL-C levels when compared with the VAP method at the postprandial state. The median total errors of LDL-C_V_ and LDL-C_MH_ ranged from 2.6 and 6.5 at the postprandial states (Figures 3B,C; Supplementary Table 1), which indicated that Vujovic and Martin–Hopkins formulas could overestimate LDL-C levels when compared with the VAP method at the postprandial state. The median total errors of postprandial LDL-C_S_ were small, i.e., 1.0 (−7.5, 8.5) and −0.3 (−10.1, 10.9) at 2 and 4 h, respectively (Figure 3D; Supplementary Table 1).

### Percentage of the accuracy of estimated low-density lipoprotein cholesterol

The accuracy of calculated LDL-C levels by formulas was considered as the percentage of the total error between −12 and 12% when compared with LDL-C_VAP_ levels. The Friedewald formula showed the highest accuracy, 80.8%, at the fasting state, followed by Sampson, Martin–Hopkins, and Vujovic formulas (Figure 3E). The Sampson formula showed the highest accuracy, 80.8%, at 2 h postprandially, followed by Friedewald, Martin–Hopkins, and Vujovic formulas (Figure 3E). At 4 h after a daily meal, the Martin–Hopkins formula and Sampson formula showed higher accuracy than Vujovic and Friedewald formulas (Figure 3E).

## Discussion

This is the first study to compare the calculated LDL-C levels by different formulas to LDL-C_VAP_ levels at both the fasting and postprandial states in Chinese subjects. We found that the calculated LDL-C_F_ levels showed the highest correlation to LDL-C_VAP_ and accuracy in evaluating fasting LDL-C levels, while the Sampson formula showed the highest accuracy at the postprandial state. Therefore, the Friedewald and Sampson formulas seemed to be a better choice to calculate fasting and postprandial LDL-C levels, respectively, in Chinese subjects.

Similar to the postprandial change in LDL-C_CM_ levels, LDL-C_VAP_ levels significantly decreased at 2 and 4 h after a daily meal in this study, which was different from the results reported by Hu et al. (22) who measured lipid profiles by enzymatic- and NMR-based methods in 87 Chinese subjects and reported that there was no significant reduction in LDL-C levels and LDL particles determined by NMR after a daily meal. However, they found cholesterol content in large LDL particles that significantly decreased at 2 and 4 h compared to the fasting one (22). American researchers compared lipid profiles detected by the VAP method between 10,135 fasting and 5,262 non-fasting (<8 h since last meal) subjects, and found significantly lower LDL-C levels and LDL particles in non-fasting subjects, although percent differences in these parameters were small (6). Chinese subjects showed a more obvious reduction in LDL-C levels at 2 and 4 h postprandially, for example, about 18% after a daily meal and 28% after a high-fat meal (18, 23). Moreover, considering that ultracentrifugation is the reference method for LDL-C measurement, and the VAP method is rapid ultracentrifugation, the reduction of LDL-C levels after a daily meal cannot be ignored, especially in Chinese subjects.

It was reported that LDL-C_CM_ levels were higher than LDL-C measured by NMR in Chinese subjects with different diseases (22). In this study, both the LDL-C_VAP_ levels and LDL-C levels calculated by formulas were lower than LDL-C_CM_ levels at both the fasting and postprandial states, which prompted us to pay more attention to the difference between LDL-C levels calculated by formulas and LDL-C_VAP_ levels.

The Friedewald formula is recommended to calculate LDL-C levels when TG levels are not very high (8). In this study, there was no subject with fasting TG ≥ 4.5 mmol/l, which may contribute to the strongest correlation between LDL-C_F_ and LDL-C_VAP_ at the fasting state. However, the TG/VLDL ratio varies with TG increasing at the fasting and non-fasting states, which decreases the accuracy of the Friedewald formula in LDL-C estimation. It is worth noting that the lowest accuracy was found in LDL-C_F_ at 4 h postprandially when TG reached the peak level. Therefore, other formulas were proposed for higher accuracy when TG increased, especially at the postprandial state.

Compared to the stable ratio of TG/VLDL-C in the Friedewald formula (2.2 for mmol/l or 5.0 for mg/dl), those in the Vujovic and Martin–Hopkins formulas were changed (9, 10). The ratio in the Vujovic formula was still fixed, but relatively greater, presenting as 3 for mmol/l or 6.85 for mg/dl (9). Its accuracy had been demonstrated in whole TC, TG, and LDL-C ranges (9). With TG levels increased, the postprandial correlation coefficients between LDL-C_V_ and LDL-C_VAP_ were stronger than the fasting state, and higher than those of the other three formulas. However, the accuracy of the Vujovic formula seemed to be relatively low, especially at the fasting state and 2 h postprandially, although it increased after a daily meal, and seemed to be better than Friedewald formula at 4 h after a daily meal. The low accuracy may be resulted from its overestimation of LDL-C compared to LDL-C_VAP_.

The ratio of TG and VLDL-C in the Martin–Hopkins formula becomes complicated and dependent on TG and non-HDL-C levels, varying from 3.1 to 11.9 (for mg/dl) (10). With TG increasing and non-HDL-C decreasing, it elevates correspondently. The accuracy of Martin–Hopkins formula was moderate on the whole, but the difference in accuracy between the fasting and postprandial states was small, which was consistent with the findings of Sathiyakumar et al., which found that the Martin–Hopkins formula was less affected by diet than the Friedewald formula (7). However, the complexity of the Martin–Hopkins ratio could reduce the convenience in clinical practice to a certain extent. After all, clinicians cannot remember so many numbers.

Other than the Friedewald, Vujovic, and Martin–Hopkins formulas, the novel Sampson formula uses higher-order mathematical terms in the form of a bivariate quadratic equation that should better reflect the amount of TG in the core of the lipoproteins (24). The Sampson formula is based on the data of 8,656 American adults with a high frequency of hypertriglyceridemia, and it was confirmed to be suitable for LDL-C calculation of samples with TG over 9 mmol/l (11), which may contribute to the highest accuracy at the postprandial states after a daily meal. Actually, at the beginning of the establishment of the Sampson formula, a comparison was made between fasting and non-fasting samples, which suggested that this formula was also applicable to non-fasting samples (11).

The LDL particles could be divided into different subfractions according to their size. The size of LDL particles had been suggested as a reliable assessment of atherogenicity (25). The subfractions of LDL particles at the postprandial states were reported to be lower by a different degree than those in fasting states (6). This may contribute to the Friedewald and Sampson formulas being the best choice for fasting and postprandial states, respectively.

This study is associated with several limitations. First, the sample size in this study was small compared to other clinical studies (7). Second, there were 46.2% of subjects got a statin history which may cause variation with those without statin use. Third, we analyzed our subjects as a whole other than stratified analysis, which may make a more precise result.

## Conclusion

In conclusion, among four formulas, the Friedewald formula was recommended to calculate fasting LDL-C, while the Sampson formula seemed to be a better choice to calculate postprandial LDL-C levels in Chinese subjects.

## Data availability statement

The raw data supporting the conclusions of this article will be made available by the authors, without undue reservation.

## Ethics statement

The studies involving human participants were reviewed and approved by Ethics Committee of The Second Xiangya Hospital of Central South University. The patients/participants provided their written informed consent to participate in this study.

## Author contributions

All authors have accepted responsibility for the entire content of this manuscript and approved its submission.

## Funding

This study was supported by grants from the National Natural Science Foundation of China (Nos. 81270956 and 81470577 to LL), the Natural Science Foundation of Hunan Province (2022JJ70136), and the Project of the Health Commission of Hunan Province, China (No. 202203012938 to LL).

## Conflict of interest

The authors declare that the research was conducted in the absence of any commercial or financial relationships that could be construed as a potential conflict of interest.

## Publisher's note

All claims expressed in this article are solely those of the authors and do not necessarily represent those of their affiliated organizations, or those of the publisher, the editors and the reviewers. Any product that may be evaluated in this article, or claim that may be made by its manufacturer, is not guaranteed or endorsed by the publisher.
