# Supplementary material for: The accuracy of four formulas for LDL-C calculation at the fasting and postprandial states
Source: Front Cardiovasc Med. 2022 Aug 18;9:944003. doi: 10.3389/fcvm.2022.944003 (PMC9433804; doi:10.3389/fcvm.2022.944003)
Supplement: Supplementary file 1 [file Table_1.pdf]

Table S1: The percentage difference between calculated and VAP measured LDL-C.

Formula	mLDL0	mLDL2	mLDL4
Friedewald	0.0(-3.8,6.0)	-3.9(-14.1,2.4)	-9.9(-15.3,0)
Vujovic	11.2(3.2,18.9)	6.1(-0.6,16.0)	6.5(-3.4，16.6)
Martin/Hopkins	7.0(-2.5,15.3)	4.6(-3.4,15.5)	2.6(-8.2,14.1)
Sampson	3.4(-1.7,10)	1.0(-7.5,8.5)	-0.3(-10.1,10.9)
