# Supplementary material for: The accuracy of four formulas for LDL-C calculation at the fasting and postprandial states
Source: Front Cardiovasc Med. 2022 Aug 18;9:944003. doi: 10.3389/fcvm.2022.944003 (PMC9433804; doi:10.3389/fcvm.2022.944003)
Supplement: Supplementary file 2 [file Image_1.pdf]

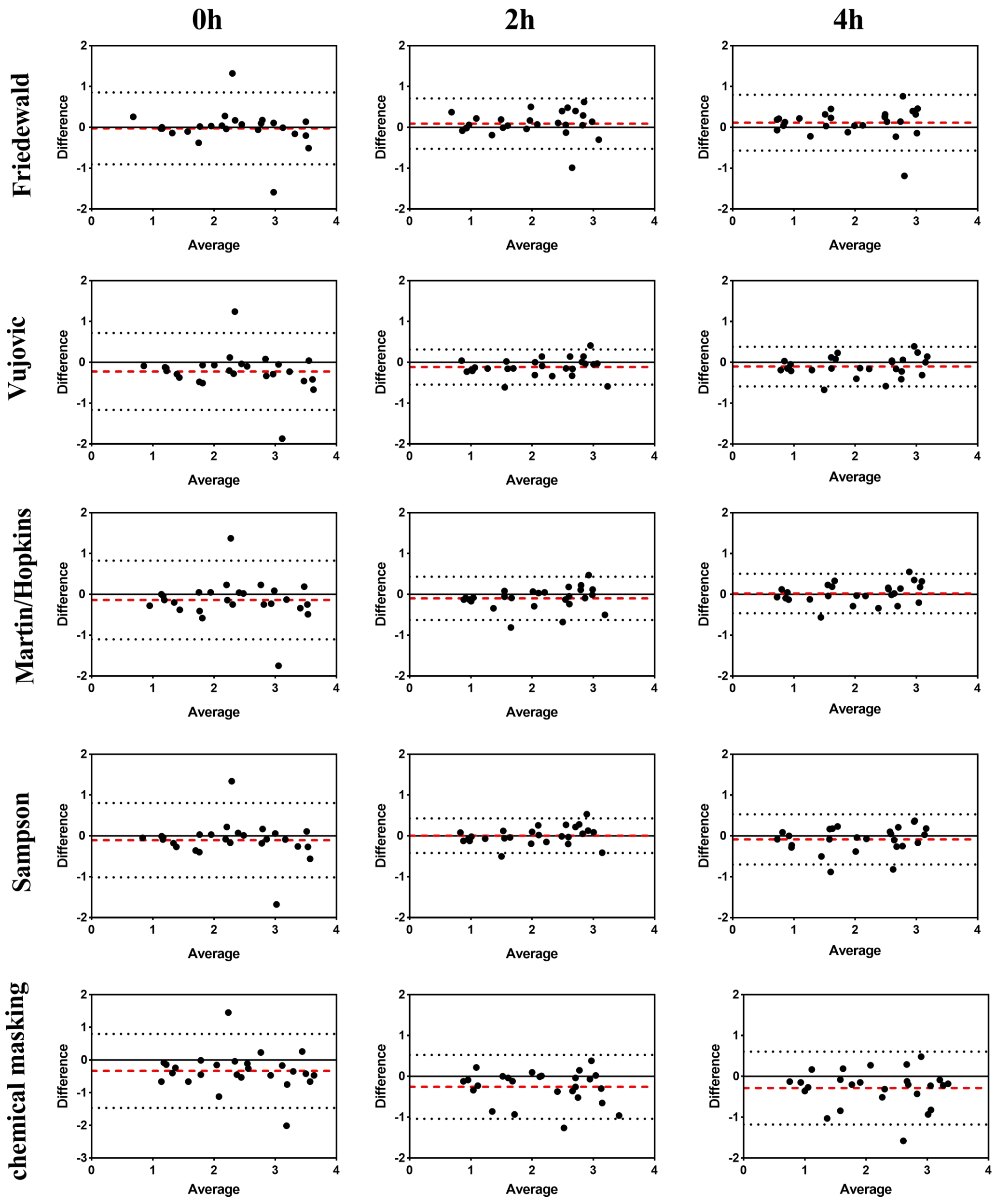

Figure S1 The Bland-Altman plots of the bias between LDL-C_{VAP} and calculated LDL-C by different formulas as well as LDL-C_{CM}.
